# An objective metric of individual health and aging for population surveys

**DOI:** 10.1186/s12963-022-00289-0

**Published:** 2022-03-31

**Authors:** Qing Li, Véronique Legault, Vincent-Daniel Girard, Luigi Ferrucci, Linda P. Fried, Alan A. Cohen

**Affiliations:** 1 School of Economics and Management, Xinjiang University, 666 Shengli Road, Urumqi, 830046 China; 2 PRIMUS Research Group, Department of Family Medicine, University of Sherbrooke, 3001 12e Ave N, Sherbrooke, QC J1H 5N4 Canada; 3 Translational Gerontology Branch, Longitudinal Studies Section, National Institute on Aging, National Institutes of Health, MedStar Harbor Hospital, 3001 S. Hanover Street, Baltimore, MD 21225 USA; 4 Mailman School of Public Health, Columbia University, 722 W. 168th Street, New York, NY R140810032 USA; 5 Research Center on Aging, 1036 Belvédère S, Sherbrooke, QC J1H 4C4 Canada; 6 Research Center of Centre Hospitalier Universitaire de Sherbrooke, 3001 12e Ave N, Sherbrooke, QC J1H 5N4 Canada

**Keywords:** Physiological dysregulation, Biomarkers, Mahalanobis distance, Population composition, Allostatic load, Self-assessed health

## Abstract

**Background:**

We have previously developed and validated a biomarker-based metric of overall health status using Mahalanobis distance (DM) to measure how far from the norm of a reference population (RP) an individual’s biomarker profile is. DM is not particularly sensitive to the choice of biomarkers; however, this makes comparison across studies difficult. Here we aimed to identify and validate a standard, optimized version of DM that would be highly stable across populations, while using fewer and more commonly measured biomarkers.

**Methods:**

Using three datasets (the Baltimore Longitudinal Study of Aging, *Invecchiare in Chianti* and the National Health and Nutrition Examination Survey), we selected the most stable sets of biomarkers in all three populations, notably when interchanging RPs across populations. We performed regression models, using a fourth dataset (the Women’s Health and Aging Study), to compare the new DM sets to other well-known metrics [allostatic load (AL) and self-assessed health (SAH)] in their association with diverse health outcomes: mortality, frailty, cardiovascular disease (CVD), diabetes, and comorbidity number.

**Results:**

A nine- (DM9) and a seventeen-biomarker set (DM17) were identified as highly stable regardless of the chosen RP (e.g.: mean correlation among versions generated by interchanging RPs across dataset of *r* = 0.94 for both DM9 and DM17). In general, DM17 and DM9 were both competitive compared with AL and SAH in predicting aging correlates, with some exceptions for DM9. For example, DM9, DM17, AL, and SAH all predicted mortality to a similar extent (ranges of hazard ratios of 1.15–1.30, 1.21–1.36, 1.17–1.38, and 1.17–1.49, respectively). On the other hand, DM9 predicted CVD less well than DM17 (ranges of odds ratios of 0.97–1.08, 1.07–1.85, respectively).

**Conclusions:**

The metrics we propose here are easy to measure with data that are already available in a wide array of panel, cohort, and clinical studies. The standardized versions here lose a small amount of predictive power compared to more complete versions, but are nonetheless competitive with existing metrics of overall health. DM17 performs slightly better than DM9 and should be preferred in most cases, but DM9 may still be used when a more limited number of biomarkers is available.

**Supplementary Information:**

The online version contains supplementary material available at 10.1186/s12963-022-00289-0.

## Background

A key challenge in the study of population health is the operationalization of a metric for global health status. In addition to potential clinical use at the individual level, such a metric would serve many purposes at the population level. It could serve as a control/adjustment variable, similar to how socioeconomic status and age are adjusted for in many epidemiological studies. It could serve as a short-term or intermediate outcome for interventions, either clinical or policy. It could be used by diverse fields ranging from health economics to sociology, demography, epidemiology, and clinical research. One approach to this problem has been using subjective metrics of global health such as self-reported health. However, subjective perception of health is conditioned by cultural or social norms as well as by medical diagnosis and access to health-care resources [1]. Thus, unless a subjective component is a main dimension to be addressed, it may be preferable to use objective health metrics that tend to be more stable [2], although specific criteria for their construction is still a matter of discussion.

A major challenge is that health is unquestionably multidimensional, and summarizing information from different indicators into a single index is not a straightforward problem. Defining the dimensions is challenging and has not yet been the study of rigorous study, to our knowledge. Various metrics of comorbidity, multimorbidity and frailty have been proposed in the literature [3–5], though most of them show limited variation among healthy younger and middle-aged adults because they are based on elements that only occur late in life. In this context, the deficit accumulation approach to frailty, based on a simple count of potential health deficits present in an individual, is particularly attractive because it is relatively robust to the precise choice of deficits in the list and health deficits can thus identify a wide range of severities, some of which are manifested even in younger individuals [6, 7]. But despite the wide use of metrics based on deficit accumulation, a standardized version has yet to be developed [8].

On the other hand, there are biomarker-based metrics that attempt to integrate the signal of multiple aspects of health. Perhaps the best-known of these is allostatic load [9]. Allostatic load is based on the theory that chronic stress can leave physiological sequalae that can be measured by creating a metric of common biomarkers linked to appropriate physiological systems: neuro-endocrine stress (cortisol, epinephrine, norepinephrine), metabolic markers (blood pressure, lipid profiles, glucose metabolism, obesity metrics), as well as a few additional biomarkers (inflammatory markers, DHEA-S, IGF-1, etc.) [10, 11]. However, allostatic load is challenging because it is conceptualized based on circular reasoning: the proxy metrics are chosen because of their known association with health and aging, so it is unsurprising the sum does as well [12]. Because it is often operationalized as a count of how many of the factors exceed clinical bounds, measures of allostatic load end up resembling comorbidity metrics in many ways, though the latter are generally not biomarker based.

Recently, our lab group has developed an alternative biomarker-based metric of physiological dysregulation based on a statistical distance (specifically, Mahalanobis distance) among biomarkers [13]. The idea is that a population average is an approximation of a homeostatic state, and that deviations from this multivariate biomarker average represent dysregulation and thus should increase with age and predict poor health state. Indeed, we have shown that dysregulation rates increase with age within individuals, and predict multiple health outcomes (mortality, frailty, various chronic diseases) after controlling for age [14, 15]. A lack of sensitivity to precise biomarker choice, and an increasing signal with more biomarkers confirm a complex systems interpretation of dysregulation as an emergent property of physiological regulatory networks [16]. Results can be replicated in many human populations [13, 14, 17–27] and even in captive primates [28] and wild birds [29]. Lastly, dysregulation can be measured either globally or by specific physiological system [30], opening up the possibility for much more detailed characterization of health state.

The dysregulation approach presents a number of clear advantages. All variables are left continuous, so there is no information loss due to categorization. The scale from 0 to infinity is appropriate for measuring dysregulation. Because it uses distances from the mean of each biomarker rather than absolute levels, it agrees with theory on biological homeostasis, which suggests that intermediate values of individual biomarkers should generally be optimal, and with evidence that variance increases with dysregulation [31, 32]. The Mahalanobis distance also incorporates the correlation structure of the variables, appropriately down-weighting redundancy among biomarkers. The insensitivity to biomarker choice means that it can be easily applied in existing datasets, can be applied in clinical contexts, and can be applied cheaply without requiring fancy, cutting-edge biomarkers. Importantly, it avoids the circularity problems present with allostatic load and metrics of biological age: the biomarkers are not selected based on correlations with age or health state, and there is no required calibration with age or health state, so the signal is an independent indicator of physiological state.

Nonetheless, some of these same advantages also present challenges. First, the possibility to use nearly any broad combination of biomarkers means that there is no standard version, and that values from one study cannot be compared directly to those from another. Second, while the approach works in every human population tested, differences in biomarker levels and correlations across populations mean that separate calibration (calculation of the mean vector and variance–covariance matrix) is required for each population. This poses problems for small studies (e.g. in clinical research) where the sample is too small to provide a robust estimation of these parameters. Third, the combination of these issues means that there are technical challenges for potential users who are less statistically inclined and would like a simple recipe.

Here, we present a standardized version of a biomarker-based global health metric that overcomes these problems. Specifically, we provide a clear methodology and rationale for choosing a subset of biomarkers that provide a strong signal, are readily available in most contexts, and can be calibrated across populations, not just within. We demonstrate the stability of the metric and its predictive power for health outcomes compared to widely used metrics: self-assessed health (SAH) and allostatic load (AL). We call the metric “DSign” for Dysregulation Signature, and propose a principle version based on 17 biomarkers and a secondary version based on 9 biomarkers, for cases in which all 17 may not be available. All biomarkers in both versions are standard clinical markers that can be readily measured in almost any setting for a very reasonable cost (e.g. < $1/marker).

## Methods

### Datasets

To construct our standard DM versions, we used data from two longitudinal cohort studies and one cross-sectional survey (see Table 1 for details): the Baltimore Longitudinal Study of Aging (BLSA), Invecchiare in Chianti (InCHIANTI), and the National Health and Nutrition Examination Survey (NHANES). BLSA, one of the world's longest studies of aging in humans, is composed of community-dwelling adults in the Baltimore and Washington DC areas aged 21–96 [33]. A 2003 re-design of methodology was tailored to improve the inference for systems-level questions [34], and we use data on 1205 individuals from after this date. InCHIANTI is a prospective population-based study of 1156 adults aged 65–102 and 299 aged 20–64, randomly selected using multistage stratified sampling from two towns in Tuscany, Italy [35]. We used data from baseline (1998–2000) and three follow-ups (2001–2003, 2005–2006, and 2007–2008). NHANES is a continuous cross-sectional stratified survey designed to be representative of the US population. Data are updated approximately every year and are made available freely (Centers for Disease Control and Prevention of the U.S. Department of Health and Human Services; http://www.cdc.gov/nchs/nhanes.htm). We used individuals aged 20 years or older from the waves 1999–2000, 2001–2002, 2003–2004, 2005–2006, 2007–2008, and 2009–2010, which have been described in detail elsewhere [36]. Unless specified otherwise, all three training datasets were kept separated to allow comparison across them.

**Table 1:** Characteristics of study populations (at first visit)

Characteristic	BLSA	InCHIANTI	NHANES	WHAS
	*n* = 1139	*n* = 1252	*n* = 17,379	*n* = 1067
Age (years)				
Mean ± SD	64.6 ± 13.8	68.2 ± 15.5	49.4 ± 19.0	77.1 ± 6.8
Range (min–max)	26.4–99.3	21.3–98.4	20–85	65.8–100.3
Female (%)	549 (48.2)	694 (55.4)	9073 (52.2)	1067 (100.0)
Race (white, %)	728 (63.9)	1252 (100.0)	8768 (50.5)	801 (75.1)
Education (years), mean ± SD	17.0 (2.6)	7.2 (14.5)	–	10.7 (3.8)
4-m walking time (s)^a^, mean ± SD	–	4.1 ± 2.8	–	9.8 ± 10.0
MMSE score^b^, mean ± SD	–	25.9 ± 3.7	–	26.5 ± 3.0
Self-assessed health				
1 (%)—highest perceived health	–	159 (13.3)	–	25 (4.2)
2 (%)	–	640 (53.7)	–	88 (14.9)
3 (%)	–	323 (27.1)	–	188 (31.9)
4 (%)	–	57 (4.8)	–	199 (33.7)
5 (%)—lowest perceived health	–	13 (1.1)	–	90 (15.3)
Allostatic load				
Mean ± SD	–	2.6 ± 1.8	–	2.1 ± 1.6
Range (min–max)	–	0–11	–	0–9

^a^We used the best performance of two attempts to walk four meters at usual pace

^b^Raw MMSE scores (ranging from 0 to 30) were used, higher scores indicating better cognition

Validation of our standard DM versions was performed with the Women’s Health and Aging Study (WHAS). WHAS is a population-based prospective study of community-dwelling women drawn from eastern Baltimore City and Baltimore County, originally consisting of two separate studies: WHAS I, which includes 1002 women aged 65 + among the 1/3 most disabled in the population [37], and WHAS II, which includes 436 women aged 70–79 among the 2/3 least disabled [38].

### Variables

Thirty-one biomarkers were available in all datasets in sufficient sample sizes (see Additional file 1: Table S1 and Fig. S1): hemoglobin, hematocrit, red cell distribution width (RDW), mean corpuscular hemoglobin (MCH), mean corpuscular hemoglobin concentration (MCHC), red blood cell count (RBC), platelets, white blood cells (WBC), basophil percentage (BASO%), lymphocyte percentage (LYM%), monocyte percentage (MONO%), neutrophil percentage (NEUT%), ferritin, glucose, calcium, chloride, sodium, potassium, vitamin B12, folate, total cholesterol, triglycerides, high density lipoprotein (HDL), albumin, alkaline phosphatase (ALKP), total proteins, gamma-glutamyl transferase (GGT), lactate dehydrogenase (LDH), uric acid, alanine transaminase (ALT), aspartate transaminase (AST).

Most health outcomes measures were only available for InCHIANTI and WHAS, and specific available data varied substantially between the two: frailty was available longitudinally in WHAS but only cross-sectionally in InCHIANTI, whereas comorbidities (specific diagnoses and count) were available cross-sectionally for WHAS but longitudinally for InCHIANTI. Specific comorbidities included diabetes and cardiovascular disease (CVD). In InCHIANTI, we used the same definitions of CVD and diabetes as previously described [30], with the following modification: we attributed a score of 1 to “possible” (0.5) scores. Sensitivity analyses previously showed that similar results are obtained by treating these possible diagnoses as absence or presence of the disease [15]. For frailty, we used the number of Fried’s frailty criteria [5] (from 0 to 5), rather than a dichotomous outcome, to increase our statistical power. Details on the number of comorbidities can also be found elsewhere [30].

Covariates for regression models included age (modelled as flexible cubic basis spline with the bs function from the fda package, using five degrees of freedom in InCHIANTI and four in WHAS), sex (InCHIANTI only), measures of physical and cognitive functions (four-meter walk time and Mini Mental State Examination [MMSE] score), and measures of socioeconomic status. Time to walk four meters was measured by asking subjects to walk 4 m at their usual pace, using a cane or walker if needed. We used the best performance (time in seconds) of two attempts. Time to walk four meters was available at all visits for InCHIANTI, but only at first visit for WHAS, and for WHAS I only (the 1/3 most disabled in the population). We used the raw score of the Mini Mental State Examination (MMSE), which measures global cognitive function with scores ranging from 0 to 30, higher scores indicating better cognition [39]. MMSE score was available at all visits for InCHIANTI, but only at first visit for WHAS. Socioeconomic variables included education level (in years) for InCHIANTI, and race, income, and education level (in years) for WHAS.

For SAH in InCHIANTI, participants answered the question: “How would you evaluate your current health? How do you feel now?” with scores from 1 (“very poor”) to 5 (“very good”). In WHAS, SAH was recorded as “perceived health condition” from 1 (“excellent”) to 5 (“poor”). To facilitate comparison, we used inverted SAH scores for InCHIANTI, such that lower scores indicate better health and higher scores worst health, mirroring DM.

### Distance-based metric of physiological dysregulation

To calculate our dysregulation score (DM), we consider individuals as points in a multi-dimensional biomarker space, where each biomarker is an axis of the space. DM defines a reference population (RP) whose centroid approximates "the ideal state", and then calculates the Mahalanobis distance to the centroid for each individual, according to Eq. 1 [40]:

1 $$D_{{\text{M}}} \left( x \right) = \sqrt {\left( {x - \mu } \right)^{T} \sum^{ - 1} \left( {x - \mu } \right)}$$

where $x$ is a vector of simultaneously observed values for the biomarkers, *μ* is the equivalent-length vector of means for each biomarker in the RP, and $\sum$ is the variance–covariance matrix of the biomarkers in the RP. Unless specified otherwise, each dataset served as its own RP in DM calculation. We also constructed a composite RP (hereinafter referred to as “composite RP”) with an equal number of subjects (*n* = 1138) from each of the three training datasets (BLSA, InCHIANTI, and NHANES; 3414 subjects in total).

Before DM calculation, all variables are transformed as necessary (log or square root) to approach normality. For each biomarker, a single best transformation was identified across datasets. The Mahalanobis distance can become unreliable when the scales of the variables differ; we thus standardize each biomarker with respect to the mean and standard deviation of the RP. Because it is approximately log-normally distributed, we used the logarithm of DM in subsequent analyses.

In calculating DM, we do not give any special weight to any of the biomarkers over and above the weights implicit in the covariance matrix. Although certain biomarkers are well-known to be important for certain diseases or physiological systems (e.g. glucose for diabetes), there is no consensus that one biomarker is more important for general health than another; subjective weighting of individual variables could thus introduce a bias in the metric.

### Selection of variables for candidate standard DM sets

We used a multistep approach in order to select biomarkers that respond best to the following criteria: (1) consistency of mean biomarker levels within and across populations; (2) stability of DM across various RPs; (3) biological signal, as measured by concordance with DM calculated using the full set (i.e. 31 biomarkers; DM31); (4) availability of biomarkers in clinical/research contexts; (5) diversity of physiological systems represented; and (6) redundancy among biomarkers. The detailed approach that led to our final sets of biomarkers can be found in Additional file 1, but, briefly, here are the four steps we followed:

1. We pre-selected 22 biomarkers by excluding those whose mean levels for various demographic subsets (e.g. males, females, young, etc.) differ across datasets or lie outside clinical bounds (criterion 1, see Additional file 1: Fig. S1).
2. We generated all possible combinations of 5 and 10 biomarkers among the 22 pre-selected ones, for which we calculated (a) correlations between DM that uses the composite RP versus each respective dataset as its own RP (see Additional file 1: Table S2, “RP stability” columns, and Additional file 1: Fig. S2), and (b) the correlation with DM that uses all 31 potential markers (DM31; see “Signal” columns in Additional file 1: Table S2). Here we aimed at identifying sets that were correlated as closely as possible with DM31 (criterion 3) while having greater robustness to RP choice (criterion 2).
3. We used subjective consideration to construct two suites of biomarkers based on the quantitative analysis performed in step 2, as well as criteria 4 and 5: a 9-biomarker set (DM9) composed of MCH, RDW, platelets, RBC, hemoglobin, WBC, BASO%, HDL, and LYM%; and a 17-biomarker set (DM17) composed of the same biomarkers as DM9, in addition to GGT, AST, ALKP, albumin, total proteins, calcium, potassium, and vitamin B12. The precise number of biomarkers included in each suite (9 and 17) was an arbitrary choice; it was rather guided by the aim to optimize criteria 2–5. The choice of two different suites, however, came from a wish to provide a shorter suite that would be more readily available in various contexts.
4. We evaluated redundancy (criterion 6) among biomarkers included in the suites selected in step 3 by looking at pairwise correlations (Additional file 1: Fig. S6); however, we did not eliminate any biomarkers based on this criterion. In the context of Mahalanobis distance, redundancy is eliminated mathematically in the calculation, and is thus not a concern. However, a good metric is likely to have markers representing a diversity of biological signals, so it is important to generate biomarker suites that are not exclusively among redundant markers.

### Stability of candidate DM sets when calibrated via different reference populations

An important issue regarding DM calculation is the choice of the RP, which is used to calculate the centroid (the biomarker combination assumed to represent optimal health) as well as the variance–covariance matrix. While there are widely accepted normal ranges for individual biomarkers, there is no consensus on a point-wise multivariate centroid that represents optimal health status. Our previous work suggested that, while a younger and healthier RP could yield a slightly better signal, the RP should not be too demographically different from the study population [17]. The entire study population itself is generally a good approximation. While it might seem intuitive that the mean of a younger, healthier population should provide a better estimate of optimal state, many age-related changes in biomarkers may actually be compensations to other changes [41, 42], raising the possibility that age-specific RPs could be preferable. Since we cannot separate out pathological changes from compensatory changes, in practice all RPs will confound these two effects to some extent.

Our goal here was to choose biomarkers that are less sensitive to the choice of RP in order to eliminate the need to consider all these factors, and to facilitate the use of a single RP for nearly any study in nearly any context. We assessed the stability of our candidate sets within and across populations. To test for stability across RPs, we computed Pearson correlations between DM calculated using the study population as its own RP, or using another dataset as the RP. Large numbers of such analyses were compiled in correlation matrices, and correlation coefficients were averaged. To test for stability within populations, we divided each dataset into demographic subsets (by sex, age, race, education level, or marital status) and performed similar correlations, i.e. between DM calculated using a given subset as its own RP or using another subset.

### Calculation of allostatic load

AL was calculated as closely as possible to previous publications [43, 44] with the available biomarkers in InCHIANTI and WHAS. We used 12 biomarkers covering five systems/functions: systolic blood pressure, diastolic blood pressure, mean arterial pressure, and heart rate were used to represent the cardiovascular system; interleukin 6 and C-reactive protein for immune functions; HDL, low density lipoprotein, glucose, and insulin-like growth factor-1 (IGF-1) for metabolic measures; body mass index for anthropometric measures; and dehydroepiandrosterone (DHEAS) for the neuroendocrine system. We attributed one point for each biomarker in the upper quartile, but in the lower quartile for IGF-1, HDL, and DHEAS, as previously described [43, 44]. Overall, AL shares only three biomarkers with DM31 (HDL, LDL, and glucose) and only one with DM17 and DM9 (HDL). Also note that biomarkers contribute to AL only if they are too far in one direction; they contribute to DM if they are far in either direction, and thus the measures would not necessarily be redundant even if there were substantial overlap.

### Association of health metrics with mortality, frailty, and comorbidities

To assess whether our final sets of biomarkers are truly representative of physiological dysregulation, we explored their association with mortality, clinical frailty, CVD, diabetes, and the number of comorbidities, in the two datasets where the relevant information was available (InCHIANTI and WHAS). We performed analyses with DM9, DM17, and DM31, all calculated using the composite RP, as well as with AL and SAH. To make the scales of DM, AL, and SAH more comparable, we divided each score by its standard deviation (SD).

The relationship between dysregulation scores and mortality was assessed using time-to-event Cox proportional hazards models with age as the timescale (coxph function, survival package). To study the relationship with frailty criteria and the number of comorbidities we used Poisson regressions, whereas logistic regressions were used for individual chronic diseases (CVD and diabetes).

As noted above (*Variables* section), some health outcomes were available only at one timepoint, and others at multiple time points, in ways that differed across datasets. When only one timepoint was available, we performed cross-sectional analyses with the glm function. When multiple timepoints were available, we included all timepoints in Bayesian multi-level models controlling for individual identity via a random intercept (MCMCglmm package [45], see Additional File 1 for details). Because of the complex patterns of cross-sectional versus longitudinal health outcome data availability, we present all results together without distinguishing the type of analysis; the goal is not to emphasize any particular result, but to assess broad patterns of predictive power for different health indices on different health oucomes.

For each health outcome, three different models were tested: (1) models that controlled for age and sex; (2) models that controlled for age, sex, and metrics of physical and cognitive functions; and (3) models that controlled for age, sex, and socio-economic status. Analyses were performed in R-3.2.2 and codes are available upon request.

## Results

### Establishment of biomarker suites

We followed a detailed procedure (see Additional file 1: Methods section 1.5) to choose subsets of biomarkers that would provide a more stable version of DM. In particular, we first narrowed the list of 31 biomarkers down to 22 by eliminating those with means that varied greatly across datasets. Among these 22, we tested each combination of 5 or 10 biomarkers, and then evaluated the impact of including/excluding a biomarker in a combination on (a) how robust DM was to choice of the RP, and (b) how closely correlated it was with the full 31-biomarker version (Additional file 1: Table S2 and Fig. S2). For example, folate introduced a strong dependency of the signal on the reference population, probably due to fortification policies in the U.S., and was thus not retained in the final list. Other subject criteria (data availability, breadth of physiological representation) were also considered to arrive at final lists of 9 and 17 markers.

### Stability of DM9 and DM17 when calibrated via different reference populations

**Fig. 1:**
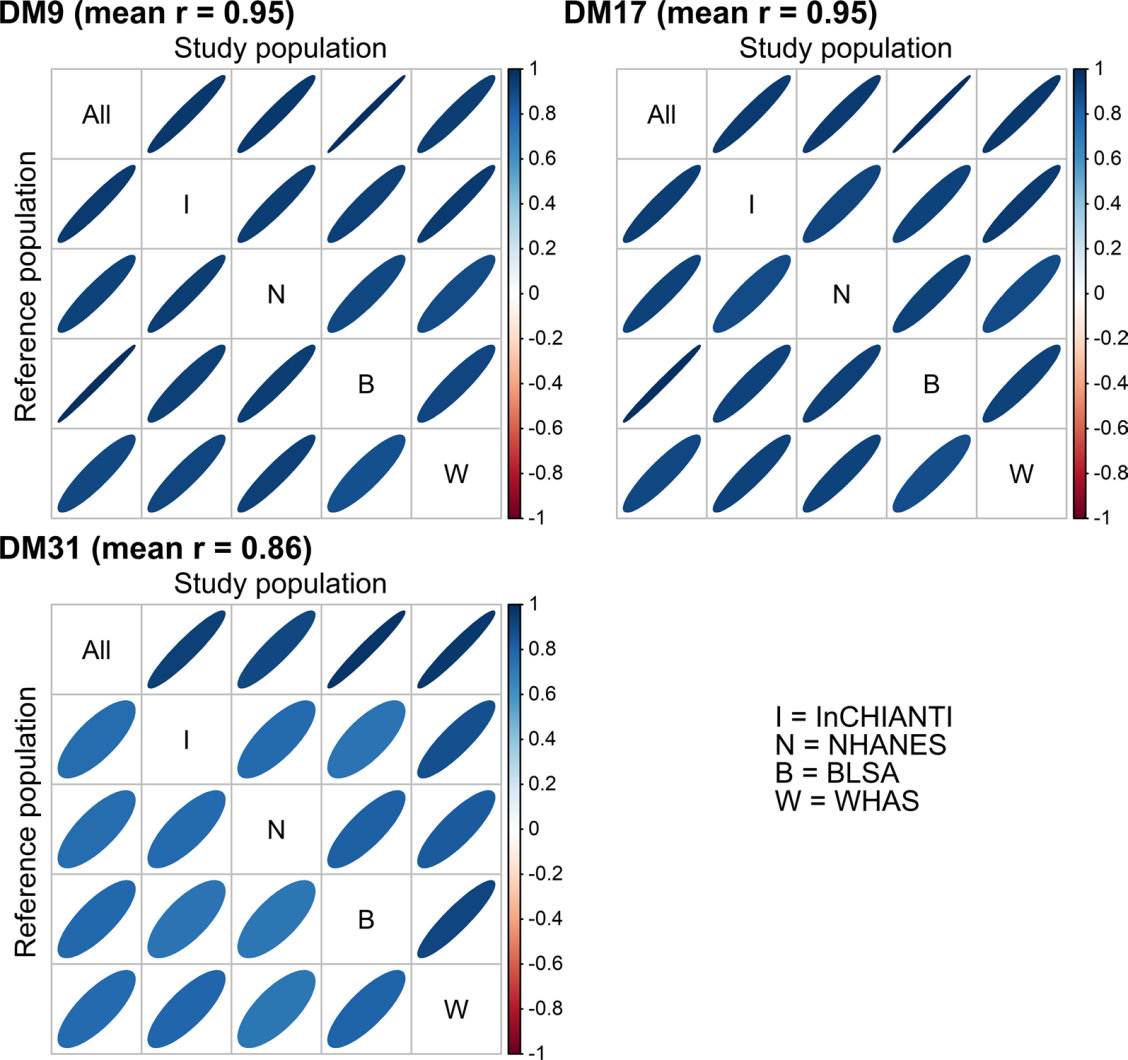
Stability of dysregulation scores across populations. For each dataset or a combined set (All), we performed correlations between dysregulation scores (DM) calculated using the study population (columns) as its own reference population or another dataset as the reference population (lines). Correlations were calculated for the three biomarker sets: 9 biomarker-set (DM9), 17-set (DM17), and the entire set (DM31). Mean Pearson correlation coefficients (r) are indicated for each set and ellipses indicate correlations visually, i.e. darker and narrower when stronger

We verified stability by asking whether DM was essentially measuring the same thing even when calibrated from different RPs, e.g., does someone who has an unusual biomarker profile for an Italian also have an unusual profile for an American? We assessed this by seeing how well two such versions of DM are correlated, and then averaging the correlations across all possible combinations. Correlations higher than 0.95 indicate high precision/stability, i.e., minimal measurement error based on calibration. DM calculated with both final sets (DM9 and DM17) proved to be highly stable, i.e. the signal did not vary substantially across various definitions of the RP (Fig. 1 and Additional file 1: Figs. S3–S5). Figure 1 shows the stability of DM signal when interchanging RPs across datasets; in other words, for any given dataset (columns), we calculated the correlations between DM calculated by using itself as the RP and DM calculated using other datasets (lines) as the RP. DM9 and DM17 are more robust than DM31 to the choice of the RP, as shown by the mean Pearson correlation coefficients of 0.95, 0.95, and 0.86, respectively obtained for DM9, DM17, and DM31. These results show that by restricting ourselves to biomarkers that vary less across different populations, we obtained a stable signal regardless of the choice of the RP. DM calculated using various demographic subsets of the study population as the RP is similarly stable (mean correlation coefficients of 0.96, 0.96, and 0.95, respectively for DM9, DM17, and DM31, see Additional file 1: Figs. S3–S5).

### Association of health metrics with mortality, frailty, and comorbidities

**Fig. 2:**
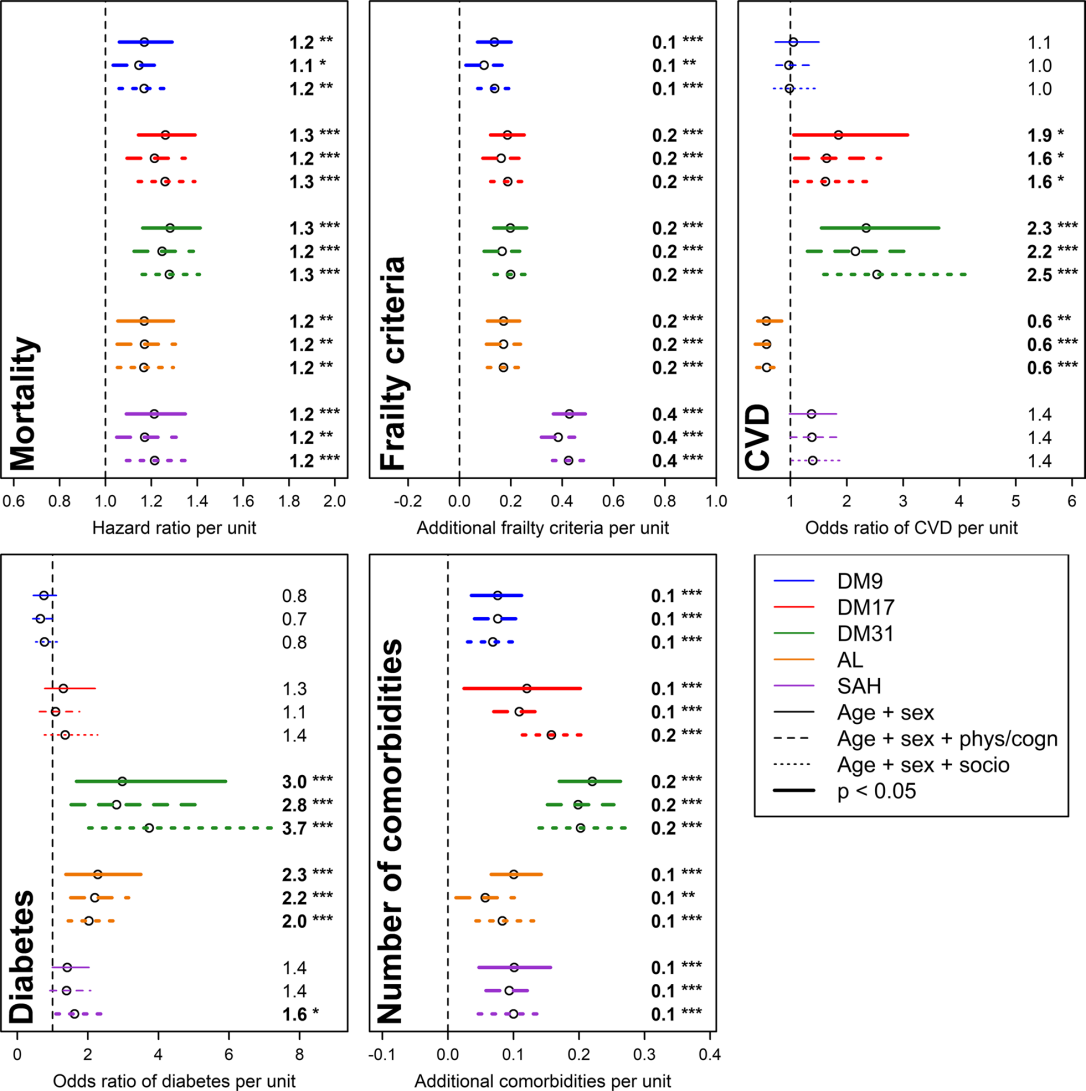
Relationships between health metrics and aging correlates in the InCHIANTI dataset. Estimations (points) together with 95% confidence intervals (CIs; segments) are plotted for mortality, the number of frailty criteria, cardiovascular diseases (CVD), diabetes, and the number of comorbidities (see text for details). Results are based on regression models adjusting for: (1) age and sex (solid lines); (2) age, sex, as well as physical and cognitive functions (dashed lines); or (3) age, sex, and socioeconomic status (dotted lines). For ease of comparison, each metric was standardized, i.e. divided by its standard deviation. Different colors refer to different health metrics and estimates are indicated on the right. Significant results are plotted in bold, with asterisks indicating the significance level (***p < 0.001; **p < 0.01; *p < 0.05). Abbreviations: AL, allostatic load; DM9, 9-set dysregulation score (DM); DM17, 17-set DM; DM31, 31-set DM; SAH, self-assessed health

**Fig. 3:**
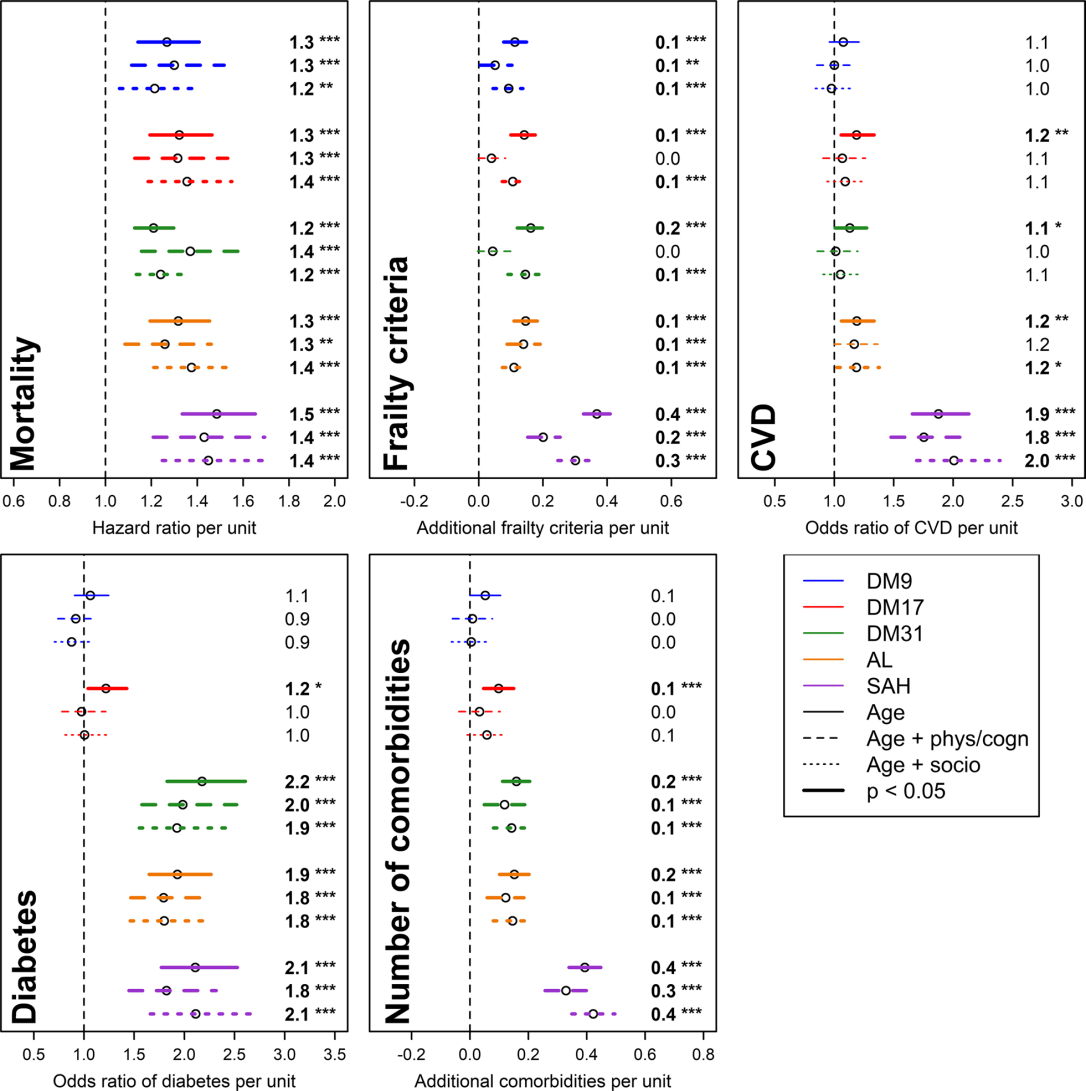
Relationships between health metrics and aging correlates in the WHAS dataset. Estimations (points) together with 95% confidence intervals (CIs; segments) are plotted for mortality, the number of frailty criteria, cardiovascular diseases (CVD), diabetes, and the number of comorbidities (see text for details). Results are based on regression models adjusting for: (1) age (solid lines); (2) age as well as physical and cognitive functions (dashed lines); or (3) age and socioeconomic status (dotted lines). For ease of comparison, each metric was standardized, i.e. divided by its standard deviation. Different colors refer to different health metrics and estimates are indicated on the right. Significant results are plotted in bold, with asterisks indicating the significance level (***p < 0.001; **p < 0.01; *p < 0.05). Abbreviations: AL, allostatic load; DM9, 9-set dysregulation score (DM); DM17, 17-set DM; DM31, 31-set DM; SAH, self-assessed health

Figures 2 and 3, as well as Additional file 1: Tables S3 and S4, show the associations of the various health metrics with health outcomes in InCHIANTI and WHAS, respectively. We added other well-known metrics of health status, namely AL and SAH, to assess how DM indices compare to existing ones that are widely used in the literature. Generally speaking, all five metrics (DM9, DM17, DM31, AL, and SAH) are competitive in their predictive ability, with some performing better in one analysis than another, but no clear “winner.” DM31 generally performed a bit better than DM17, which performed a bit better than DM9, as expected.

**Fig. 4:**
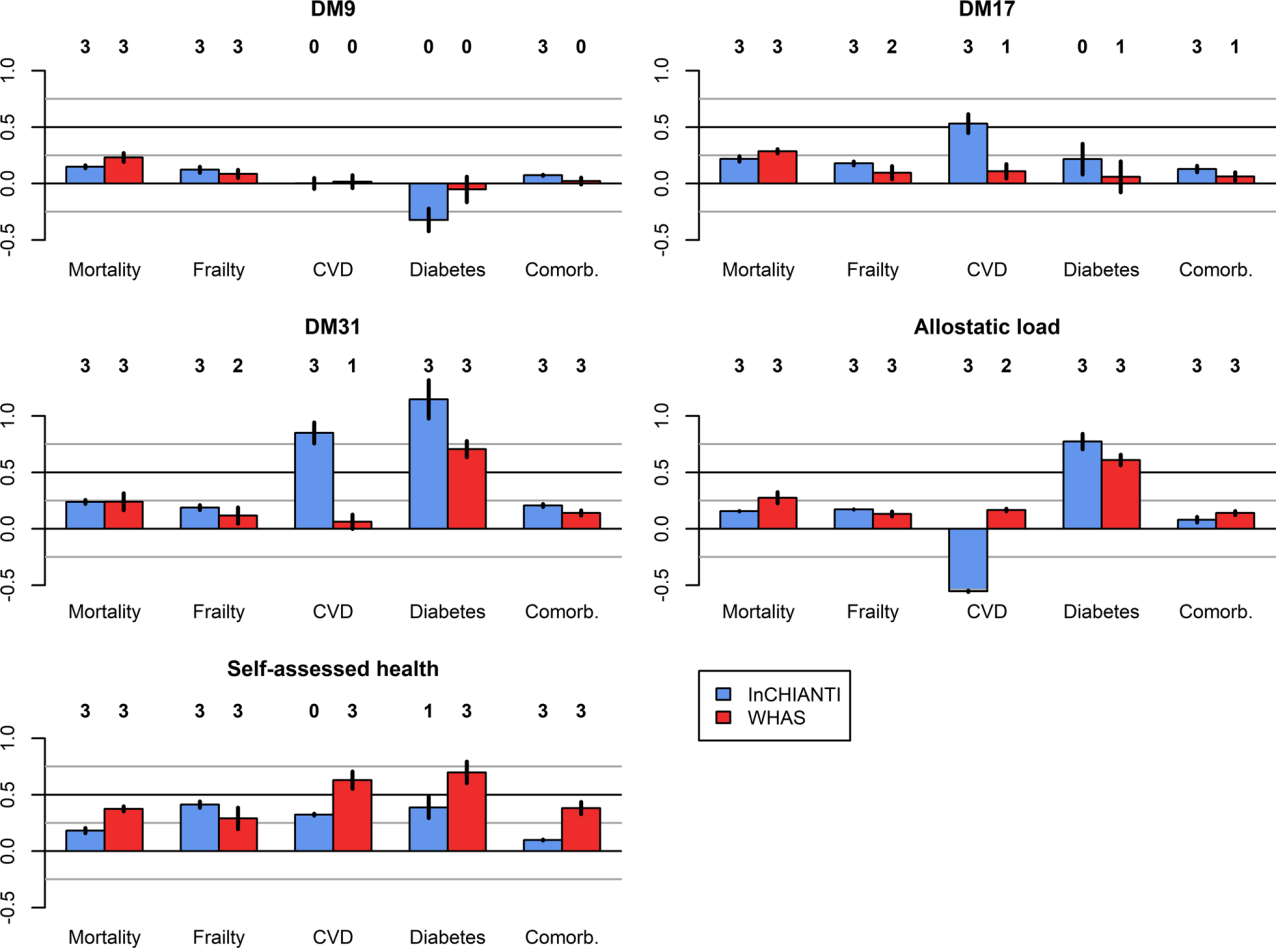
Comparison of predictive performance across health metrics for various health outcomes. Bars represent the means of estimated regression coefficients for the three different analyses performed (see Figs. 2, 3) in InCHIANTI (blue) and WHAS (red), with the corresponding 95% confidence interval. For ease of comparison across health outcomes, we used the log-hazard and log-odds ratios. Numbers above the bars indicate the number of significant associations out of three analyses. Abbreviations: Comorb., number of comorbidities; DM9, 9-set dysregulation score (DM); DM17, 17-set DM; DM31, 31-set DM

All metrics are comparable for mortality prediction (hazard ratio ranges of 1.15–1.30, 1.21–1.36, 1.21–1.37, 1.17–1.38, and 1.17–1.49, respectively for DM9, DM17, DM31, AL, and SAH, respectively); however, DM-based metrics tend to show less variation across datasets (Fig. 4).

SAH appears to be more strongly associated with frailty than the biomarker-based metrics: estimated regression coefficients were of 0.41 and 0.29, respectively for InCHIANTI and WHAS, whereas other metrics only reached ~ 0.15 (Figs. 2–3).

In InCHIANTI, DM31 appeared to perform particularly well for CVD and diabetes prediction (odds ratios ranging from 1.01 to 1.85 and from 1.93 to 3.73, respectively), likely reflecting the inclusion of metabolic-syndrome-related biomarkers in this version. Similarly, the high performance of AL for diabetes prediction (odds ratio ranging from 1.79 to 2.28) might be due to the inclusion of glucose in its calculation, as opposed to DM9 and DM17. Nevertheless, DM17 was overall a reasonable predictor of CVD (odds ratios ranging from 1.07 to 1.85, p < 0.05 in four out of six analyses; see Figs. 2–4), as opposed to DM9 (odds ratios ranging from 0.97 to 1.08, no significant association).

Prediction of the number of comorbidities is also relatively similar across metrics, with ranges of estimated regression coefficients of 0–0.08, 0.03–0.16, 0.12–0.22, 0.06–0.15, and 0.09–0.42, respectively for DM9, DM17, DM31, AL, and SAH (Figs. 2–3).

## Discussion

We have previously proposed a metric of physiological dysregulation (DM), based on statistical distance and relying exclusively on common clinical biomarkers [13]. Here we aimed to reduce the number of biomarkers used in its calculation so that DM can be used in contexts where fewer biomarkers are available (e.g. in socio-economic studies) and to propose a version of DM that is highly stable across different populations, so that it can be easily compared across studies. We had previously shown that DM’s signal increases with the number of biomarkers included, although the value of additional markers diminishes as more are added [17], and that inclusion of 10–15 is generally sufficient. Using solely biomarkers from the complete blood count, the lipid and liver panels, as well as calcium and vitamin B12, we identified and validated two DM versions: a version using 17 biomarkers and a shorter version that uses only 9 biomarkers, excluding the ones that may be slightly less common (GGT, ALKP, AST, albumin, total proteins, calcium, and vitamin B12). Nine or 17 markers may seem like a lot, but eight are measured together in the complete blood count, while HDL is highly common, and many of the liver proteins are measured together in a panel; many existing studies already have all these markers. The main advantage of the 9-marker version would be in secondary data analysis when one of the 17 markers is missing; in prospective studies, it should usually be feasible to measure all 17. Both versions proved to be highly stable across various definitions of the RP and to provide good predictions of health outcomes, though the 17-biomarker version performs slightly better for prediction. We thus propose these dysregulation signatures (“DSign”) as generalized, objective metrics of health state, with DM17 to be preferred when possible.

As expected, there was no clear “winner” among metrics of health state to predict various health outcomes. Some metrics performed better for certain outcomes or in one or the other dataset. For example, SAH performs best for predicting phenotypic frailty, an unsurprising result given that phenotypic frailty is diagnosed based on physical symptoms a patient would recognize rather than on measurement of the underlying pathology. Likewise, as expected, DM31 generally performs as well as or better than DM17 and DM9 for predicting health outcomes, particularly diabetes and CVD, which are related to some of the metabolic-syndrome-associated biomarkers that were eliminated in order to increase robustness of the signal. Nonetheless, for mortality, all metrics perform about equivalently. Interestingly, in most cases the strengths of the effects were minimally impacted by control for covariates, including socioeconomic status and markers of physical and cognitive functioning. This was true not just for versions of DM, but also AL and SAH. Potentially, this is due the underlying health state mediating the impacts of the covariates on the outcomes.

Health plays an important role in many study fields and efforts have been made in the search for robust and comparable health metrics. While many existing health metrics are good predictors of mortality, frailty and comorbidities, notably SAH, we believe it is meaningful to have an objective and continuously distributed metric of general health based on continuous variables (biomarkers). First, a continuous health metric can facilitate the estimation of the distribution of health states. Indices of health inequality can also be easily calculated with the continuous health metric. For example, the concentration index has become a standard metric to quantify income-related inequalities [46]. Strictly speaking, the concentration index is an appropriate metric of socioeconomic-related health inequality when health is measured on a ratio scale with a true zero [47]. Our health metric satisfies these requirements by definition, where a value of zero represents the ideal state of health. An application to the calculation of the concentration index was illustrated in a working paper [48]. Second, a continuous health metric facilitates the use of certain statistical tools, such as ordinary least squares or instrumental variable regression, whose consistency relies less on distributional assumptions [49]. Single biomarkers have occasionally been used as indicators of health outcome in statistical models that require a continuous health variable [50]; however, it would be preferable to summarize the information from multiple biomarkers into a single metric when measuring global health. Third, in comparison with subjective health metrics (e.g. self-reported health) or quasi-objective health metrics (e.g. composite health metrics constructed from survey questions) the health metric here could be applied more easily across different populations without being influenced by cultural differences or reporting habits. Indeed, several studies have reported differences in rating health according to gender [51, 52], ethnicity [53, 54], and age [55, 56]. Last but not least, in keeping the biomarkers continuous during the construction of the health metric, we may well avoid loss of information associated with categorization of continuous variables [57]. In cases where the study population is small or broadly representative of the population, we strongly recommend using our reference population; however, in cases where the study population is both large enough to serve as its own reference, and is highly specific (e.g. suffering from a particular disease, children, a non-industrialized tribe), we would recommend using the study population as the reference population.

Because the DSign metric is based on the notion that an “average” biomarker profile is more likely to be healthy, it is, at least in theory, sensitive to the characteristics of the reference population that is used for calibration. For example, one would not expect the mean glucose level in a population with a high prevalence of diabetes to be a good indicator of ideal level. While such concerns are indeed problematic in low dimensions (2–3 markers), as the number of markers increases, DM becomes less and less sensitive to precise estimation of the mean for each marker. This is because, in high dimensions, it becomes very improbable to be very close to the mean on all parameters, and accordingly no individual ever falls very close to the true multi-dimensional centroid; the distribution is “hollowed out.” As long as the estimated centroid falls within this hollowed out area, there is minimal impact of misestimation. In the versions of DM presented here, this concern is even less present because we have specifically sought to eliminate markers that differ in means across reference populations. This is a key criterion for stability. We nonetheless counsel caution in the interpretation of DM in cases where a population’s mean or distribution may differ markedly from the reference population.

It is important to note several limitations of this approach as well. First, we would not recommend application of this DSign metric to populations suffering from a specific disease. For example, a study on the efficacy or safety of a medication for patients on hemodialysis should not rely on DM17 or DM9 as a proxy outcome, because hemodialysis a priori represents a state of extreme dysregulation of multiple biomarkers [58], for which our standardized RP would be inappropriate without independent validation. Second, there are clearly multiple dimensions to physiological health, and any single metric is by definition a crude simplification [30, 59]. The advantages of this approach should not be used to gloss over the limitations of any such approach. Third, the advantages of this approach do not make it the best choice in all cases. For example, SAH may be a preferable representation of health state in some cases, either for practical reasons (e.g. better prediction of frailty, empirically) or theoretical reasons (e.g. a specific interest in how perception of one’s health influences outcomes). Fourth, we do not claim that the version presented here is the only valid version of DM, or necessarily the best; it is one approach among many that appears to represent a nearly optimal balance of usability, stability, and predictive value, but sophisticated users may prefer to develop their own versions based on data availability or their specific needs for these sometimes-conflicting factors. Fifth, the populations used to establish stability here, while from two continents, nonetheless represent industrialized, Western societies. Caution should be exercised applying the metric to other populations, though studies have shown that DM does work well as a health metric in several provinces in Chinese mainland, in Taiwan, and in the Tsimane horticulturalists of Bolivia [18–20, 27]. We believe it would probably apply well in most contexts, but maybe not in populations with highly specific characteristics (e.g. patients on hemodialysis).

## Conclusions

We have developed a continuous, biomarker-based, standardized, validated metric of health state. While no single metric can be universally optimal, this metric presents a number of clear advantages: simplicity of use, ease to obtain the relevant biomarkers, predictive power competitive with other well-known metrics, stability across populations, and theoretical non-circularity. For many users, it will present a substantial improvement over previously published versions of DM, notably in its standardization and stability. In contexts where blood collection is difficult to achieve, SAH appears as reasonable alternative to blood-based metrics, given its strong predictive performance in nearly all health outcomes studied here. We nonetheless strongly urge users of any generalized health metric to use caution and a nuanced interpretation, given the inherent challenges of using a single metric to measure a multi-dimensional process in a complex system.

## Supplementary Information

**Additional file 1.** Supplementary methods, tables and figures.

## Data Availability

With the exception of NHANES, the data used in these analyses cannot be freely shared due to confidentiality constraints related to human medical data, but they are all available to researchers submitting an appropriate research proposal: InCHIANTI at http://www.inchiantistudy.net/obtain_data.html, WHAS at https://jhpeppercenter.jhmi.edu/ec_proposal/login.aspx, and BLSA at http://www.blsa.nih.gov/researchers. NHANES data is available at https://www.cdc.gov/nchs/nhanes/.

## Abbreviations

AL: Allostatic load
ALKP: Alkaline phosphatase
ALT: Alanine transaminase
AST: Aspartate transaminase
BASO%: Basophil percentage
BLSA: Baltimore Longitudinal Study of Aging
DM: Mahalanobis distance (dysregulation score)
DM9: 9-Set DM
DM17: 17-Set DM
DM31: 31-Set DM
GGT: Gamma-glutamyl transferase
HDL: High density lipoprotein
InCHIANTI: Invecchiare in Chianti
LDH: Lactate dehydrogenase
LYM%: Lymphocyte percentage
MCH: Mean corpuscular hemoglobin
MCHC: Mean corpuscular hemoglobin concentration
MONO%: Monocyte percentage
NEUT%: Neutrophil percentage
NHANES: National Health and Nutrition Examination Survey
RBC: Red blood cell count
RDW: Red cell distribution width
RP: Reference population
SAH: Self-assessed health
SD: Standard deviation
WBC: White blood cells
WHAS: Women’s Health and Aging Study
