# Supplementary material for: An objective metric of individual health and aging for population surveys
Source: Popul Health Metr. 2022 Mar 31;20:11. doi: 10.1186/s12963-022-00289-0 (PMC8974028; doi:10.1186/s12963-022-00289-0)
Supplement: Supplementary file 1 — Additional file 1. Supplementary methods, tables and figures. [file 12963_2022_289_MOESM1_ESM.docx]

**An objective measure of individual health and aging for population surveys**

Qing Li, Alan A. Cohen, Véronique Legault, Vincent-Daniel Girard, Luigi Ferrucci, Linda P. Fried

1. **Supplementary Methods**
   1. **Selection of biomarkers**

**Step 1**: We made a first pre-selection based on mean levels for various population subsets (e.g. males, females, young, etc.), selecting biomarkers for which mean levels are similar across datasets and for different population subgroups, and that lie within clinical bounds (criterion 1, see Supplementary Fig. 1). This yielded a preliminary set of 22 biomarkers: hemoglobin, RDW, MCH, RBC, platelets, WBC, BASO%, LYM%, MONO%, calcium, chloride, sodium, potassium, vitamin B12, folate, HDL, albumin, ALKP, total proteins, GGT, ALT, AST.

**Step 2**: We conducted quantitative analysis on this set of 22 biomarkers to evaluate how the inclusion of each biomarker affected the performance for criteria 2-3 (stability of DM across reference populations and biological signal, respectively). To do this, we first constructed a composite RP with equal numbers of subjects (n = 1138) from each of the three training datasets. We then ran analyses on every possible combination of 10 biomarkers from among the 22, and on every combination of 5 biomarkers. For each combination, we calculated DM based on the composite RP and based on the RP of the respective dataset (BLSA, InCHIANTI, and NHANES). The Pearson correlation coefficient between these two was considered an indication of the robustness of DM to choice of reference population. We then selected the subset of combinations for which *r* > 0.95 (particularly robust), and calculated the percentage of these combinations that contained each biomarker (see Supplementary Table 2, “RP stability” columns; see also Supplementary Fig. 2). A high percentage indicates that inclusion of that biomarker in the calculation of DM is associated with greater robustness to choice of RP.

We then used the same set of biomarker combinations to calculate how well each DM version was correlated with the full DM31 version containing all potential markers. The full version is expected to have more biological signal, but to be less stable, so we were looking for a combination that is correlated as closely as possible with DM31 while having greater robustness to RP choice. For each biomarker, we calculated the average Pearson correlation coefficient with DM31 for the combinations that contain that marker. These are indicated in the “Signal” columns in Supplementary Table 2 (see also Supplementary Fig. 2). Based on these criteria, we were able to eliminate folate and monocyte counts.

**Step 3**: We used subjective consideration of criteria 4 and 5 (data availability and breadth of biomarker representation) to eliminate sodium, potassium, and chloride from the list. We also then used an integration of all criteria to subjectively construct two suites, one with 9 biomarkers that would be more readily available, and one with 17 that would provide a slightly better signal.

**Step 4**: We evaluated whether any of the biomarkers were particularly redundant with others using correlation coefficients (criterion 6, see Supplementary Fig. 6), but apart from a few exceptions, biomarkers were mostly uncorrelated or very poorly. In fact, biomarkers highly correlated are very likely to be measured together in a blood test (e.g. hemoglobin and RBC; ALT and AST). We did not eliminate any biomarkers based on the redundancy criterion.

- 1. **Details on Bayesian regression models**

Models included population- and individual-level intercept, and burn-in period, number of iterations and thinning interval were respectively set at 7000, 17,000, and 10. We used prior1 and prior2 (specified below), respectively for logistic and Poisson regressions:

$prior1= list(R = list(V = 1, fix=1),G = list(G1 = list(V = 1, nu = 0.002)))$

$prior2= list(R = list(V = 1, nu = 0.002), G = list(G1 = list(V = 1, nu = 0.002)))$

- 1. **Composite reference population**

Our online tool (<https://github.com/cohenaginglab/DM>) offers the possibility to calculate DM9, DM17, and DM31 using the RP used to validate it. It is composed of 1138 random subjects from BLSA, InCHIANTI, and NHANES datasets (3414 individuals in total), aged 20 to 99 years (mean age of 59.6 years). Nearly half (44.6%) are men.

1. **Supplementary Tables**

**Supplementary Table 1. Biomarkers used, their values by data set, and reported reference ranges.**

| **Biomarker** | **InCHIANTI** | |  | **BLSA** | |  | **NHANES** | |  | **WHAS** | |  | **Reference Range** |
| --- | --- | --- | --- | --- | --- | --- | --- | --- | --- | --- | --- | --- | --- |
|  | **Mean** | **SD** |  | **Mean** | **SD** |  | **Mean** | **SD** |  | **Mean** | **SD** |  |  |
| Hemoglobin (g/dL) | 13.8 | 1.5 |  | 13.6 | 1.4 |  | 14.2 | 1.6 |  | 12.9 | 1.2 |  | ̶ |
| Male | 14.6 | 1.3 |  | 14.2 | 1.4 |  | 15.1 | 1.3 |  | - | - |  | 13.0-17.0^1^ |
| Female | 13.3 | 1.1 |  | 13.1 | 1.2 |  | 13.4 | 1.3 |  | 12.9 | 1.2 |  | 12.3-15.7^1^ |
| Hematocrit (%) | 41 | 4 |  | 41 | 4 |  | 42 | 4 |  | 39.1 | 3.7 |  | ─ |
| Male | 43 | 4 |  | 42 | 4 |  | 44 | 4 |  | - | - |  | 38-50^1^ |
| Female | 40 | 3 |  | 39 | 3 |  | 39 | 4 |  | 39.1 | 3.7 |  | 37-46^1^ |
| Red cell distribution width (RDW, %) | 13.7 | 1.2 |  | 13.5 | 1.5 |  | 12.9 | 1.2 |  | 14.1 | 1.4 |  | 11.5-14.5^1^ |
| Mean corpuscular hemoglobin (MCH, pg/cell) | 30.6 | 2.1 |  | 30.4 | 2.1 |  | 30.5 | 2.3 |  | 30.5 | 2.1 |  | 27-34^1^ |
| Mean corpuscular hemoglobin conc. (MCHC, g/dL) | 33.7 | 1.0 |  | 33.5 | 1.2 |  | 34.0 | 0.9 |  | 33.1 | 1.2 |  | 31.0-37.0^1^ |
| Red blood cell count (RBC, millions/µL) | 4.6 | 0.5 |  | 4.5 | 0.5 |  | 4.7 | 0.5 |  | 4.3 | 0.4 |  | ─ |
| Male | 4.7 | 0.4 |  | 4.6 | 0.5 |  | 5.0 | 0.5 |  | - | - |  | 4.4-5.7^1^ |
| Female | 4.4 | 0.4 |  | 4.4 | 0.4 |  | 4.4 | 0.4 |  | 4.3 | 0.4 |  | 4.0-5.2^1^ |
| Platelet count (10^3^/mm^3^) | 235 | 63 |  | 231 | 74 |  | 267 | 70 |  | 242 | 68 |  | 130-400^1^ |
| Ferritin (ng/mL) | 123 | 123 |  | 107 | 99 |  | 119 | 153 |  | 112 | 124 |  | 50-150^2^ |
| White blood cell count (WBC, K/µL) | 6.2 | 1.7 |  | 6.0 | 3.5 |  | 7.3 | 2.5 |  | 6.3 | 2.4 |  | 4-10^1^ |
| Neutrophil count (%) | 58.4 | 8.7 |  | 54.6 | 10.4 |  | 58.7 | 9.7 |  | 60.2 | 10.1 |  | 45-75^1^ |
| Monocyte count (%) | 6.6 | 2.2 |  | 9.2 | 4.3 |  | 7.9 | 2.3 |  | 6.9 | 2.4 |  | 4-11^1^ |
| Lymphocyte count (%) | 31.3 | 8.1 |  | 32.4 | 9.9 |  | 29.9 | 8.7 |  | 29.0 | 9.1 |  | 16-46^1^ |
| Basophil count (%) | 0.5 | 0.4 |  | 0.6 | 0.3 |  | 0.7 | 0.5 |  | 0.7 | 0.5 |  | 0-3^1^ |
| Calcium (mg/dL) | 9.4 | 0.5 |  | 9.3 | 0.4 |  | 9.4 | 0.4 |  | 9.4 | 0.4 |  | 8.7-10.3^1^ |
| Chloride (mEq/L) | 106 | 4 |  | 104 | 3 |  | 103 | 3 |  | 103 | 4 |  | 98-106^1^ |
| Sodium (mEq/L) | 141 | 3 |  | 142 | 3 |  | 139 | 2 |  | 140 | 3 |  | 135-145^1^ |
| Potassium (mEq/L) | 4.2 | 0.4 |  | 4.2 | 0.3 |  | 4.0 | 0.3 |  | 4.2 | 0.4 |  | 3.5-5.0^1^ |
| Total cholesterol (mg/dL) | 215 | 42 |  | 191 | 37 |  | 200 | 43 |  | 224 | 41 |  | < 200^4^ |
| Triglycerides (mg/dL) | 128 | 77 |  | 103 | 58 |  | 145 | 125 |  | 160 | 98 |  | < 150^4^ |
| High density lipoprotein (HDL, mg/dL) | 57 | 15 |  | 59 | 17 |  | 53 | 16 |  | 55.1 | 16.2 |  | ̶ |
| Male | 53 | 14 |  | 53 | 14 |  | 48 | 14 |  | - | - |  | > 38.6^4^ |
| Female | 61 | 15 |  | 66 | 17 |  | 58 | 17 |  | 55.1 | 16.2 |  | > 50.2^4^ |
| Albumin (g/dL) | 4.3 | 0.3 |  | 4.1 | 0.3 |  | 4.2 | 0.4 |  | 4.1 | 0.3 |  | 3.5-5.0^1^ |
| Alkaline phosphatase (Alk. Phos., U/L) | 81 | 39 |  | 78 | 23 |  | 73 | 28 |  | 87.2 | 35.2 |  | 38-126^1^ |
| Total proteins (g/dL) | 7.3 | 0.5 |  | 7.1 | 0.5 |  | 7.2 | 0.5 |  | 7.0 | 0.5 |  | 6.0-8.0^1^ |
| gamma-glutamyl transpeptidase (GGT, U/L) | 26.5 | 31.6 |  | 30 | 24 |  | 31 | 46 |  | 30.5 | 35.9 |  | ̶ |
| Male | 32 | 38 |  | 33 | 24 |  | 37 | 55 |  | - | - |  | 10-48^1^ |
| Female | 22 | 24 |  | 29 | 22 |  | 25 | 35 |  | 30.5 | 35.9 |  | 10-30^1^ |
| Lactate dehydrogenase (LDH, U/L) | 344 | 75 |  | 430 | 163 |  | 135 | 34 |  | 176 | 35 |  | 95-195^1^ |
| Uric acid (mg/dL) | 5.1 | 1.4 |  | 5.3 | 1.4 |  | 5.4 | 1.5 |  | 5.6 | 1.7 |  | 3.0-7.1^1^ |
| Alanine transaminase (ALT, U/L) | 19.8 | 15.1 |  | 32.0 | 12.4 |  | 25.7 | 26.5 |  | 16.1 | 12.1 |  | 13-63^1^ |
| Aspartate transaminase (AST, U/L) | 21.0 | 9.6 |  | 28.1 | 10.6 |  | 25.7 | 19.8 |  | 19.6 | 10.9 |  | 18-40^1^ |
| Glucose (mg/dL) | 94.3 | 24.6 |  | 92.8 | 17.7 |  | 106.3 | 37.0 |  | 113.9 | 56.6 |  | 59-105^1^ |
| Folate (nmol/L) | 10.0 | 6.8 |  | 55.7 | 32.0 |  | 45.3 | 26.9 |  | 28.1 | 23.6 |  | 7-36^1^ |
| Vitamin B12 (pmol/L) | 350 | 247 |  | 472 | 270 |  | 438 | 1737 |  | 365 | 227 |  | 133-674^1^ |

^1^ Based on recommendations from the Medical Council of Canada, Objectives for the Qualifying Examination, 3rd Edition.

^2^ Based on recommendations from the Iron Disorders Institute’s Scientific & Medical Advisory Board 2010.

^3^ Based on Kratz A, Ferraro M, Sluss PM, *et al.* (2004). Case records of the Massachusetts General Hospital. Weekly clinicopathological exercises. Laboratory reference values. *N Engl J Med*, **351**(15):1548-63.

^4^ Based on recommendations from the Canadian Health Measures Survey Physician Advisory Committee 2010.

^5^ Based on recommendations from the U.S. National Cholesterol Education Program.

^6^ Based on recommendations from Hypertension Canada guidelines published in Leung, Alexander A. et al. (2017). Hypertension Canada’s 2017 Guidelines for Diagnosis, Risk Assessment, Prevention, and Treatment of Hypertension in Adults. *Can J Cardiol*, **33**(5):557-576.

**Supplementary Table 2. Criteria used for biomarker selection.**

|  |  | **10 biomarker sets** | |  | **5 biomarker sets** | |
| --- | --- | --- | --- | --- | --- | --- |
|  | **System** | **RP stability**^a^ | **Signal**^b^ |  | **RP stability**^a^ | **Signal**^b^ |
| **MCH** | OT | 0.596 | 0.701 |  | 0.344 | 0.564 |
| **RBC** | OT | 0.577 | 0.692 |  | 0.316 | 0.535 |
| **Hemoglobin** | OT | 0.576 | 0.683 |  | 0.318 | 0.515 |
| **RDW** | OT | 0.542 | 0.681 |  | 0.314 | 0.525 |
| **GGT** | LF | 0.518 | 0.667 |  | 0.275 | 0.490 |
| **Total proteins** | BOP | 0.517 | 0.668 |  | 0.290 | 0.489 |
| **Platelets** | C | 0.517 | 0.667 |  | 0.276 | 0.488 |
| **Lymphocytes** | I | 0.514 | 0.664 |  | 0.292 | 0.477 |
| **Potassium** | E | 0.514 | 0.664 |  | 0.301 | 0.478 |
| **HDL** | L | 0.511 | 0.666 |  | 0.290 | 0.477 |
| **ALKP** | LF | 0.509 | 0.668 |  | 0.308 | 0.485 |
| **Calcium** | E | 0.501 | 0.671 |  | 0.273 | 0.492 |
| **WBC** | I | 0.499 | 0.666 |  | 0.256 | 0.484 |
| **Vitamin B12** | V | 0.491 | 0.665 |  | 0.229 | 0.475 |
| **Basophils** | I | 0.468 | 0.662 |  | 0.252 | 0.470 |
| **AST** | LF | 0.466 | 0.672 |  | 0.306 | 0.498 |
| **Albumin** | BOP | 0.446 | 0.666 |  | 0.217 | 0.481 |
| **Sodium** | E | 0.395 | 0.665 |  | 0.048 | 0.478 |
| **Chloride** | E | 0.386 | 0.666 |  | 0.089 | 0.479 |
| **ALT** | LF | 0.378 | 0.667 |  | 0.003 | 0.488 |
| **Folate** | V | 0.050 | 0.657 |  | 0.000 | 0.451 |
| **Monocytes** | I | 0.028 | 0.690 |  | 0.001 | 0.515 |

^a^ To calculate how each biomarker influenced the stability of DM across different RPs, for all combinations among the 22-set, we calculated correlations between DM calculated with the study population as its own reference population, or with a combined reference population composed of 1138 individuals from each study dataset (BLSA, InCHIANTI, NHANES). The numbers in this column are the frequency of each biomarker among the combinations with a coefficient >= 0.95, such that higher values indicate this biomarker is more often present in highly stable DMs.

^b^ To assess the impact of including or excluding each biomarker on the biological signal of DM, we assessed the correlation of each combination with the full DM31 set. In this column, we present the mean Pearson correlation coefficient (*r*) for all combinations including the row’s biomarker.

Abbreviations: ALKP, Alkaline phosphatase; ALT, alanine transaminase; AST, aspartate transaminase; BOP, blood oncotic pressure; C, Coagulation; E, Electrolytes; GGT, gamma-glutamyl transferase; HDL, high density lipoprotein; I, Immune; L, Lipids; LF, Liver function; MCH, mean corpuscular hemoglobin; OT, Oxygen transport; RBC, red blood cell count; RDW, red cell distribution width; V, Vitamins; WBC, white blood cells.

**Supplementary Table 3. Relationships between health metrics and various health outcomes in the InCHIANTI dataset.**

|  | Model 1^a^ | Model 2^b^ | Model 3^c^ |
| --- | --- | --- | --- |
| **Mortality (hazard ratio with 95% CI)** | | | |
| DM9 | 1.17 (1.06, 1.29) | 1.15 (1.03, 1.27) | 1.17 (1.06, 1.29) |
| DM17 | 1.26 (1.14, 1.39) | 1.21 (1.09, 1.35) | 1.26 (1.14, 1.39) |
| DM31 | 1.28 (1.16, 1.41) | 1.25 (1.12, 1.38) | 1.28 (1.16, 1.41) |
| Allostatic load | 1.17 (1.05, 1.30) | 1.17 (1.05, 1.30) | 1.17 (1.05, 1.30) |
| SAH | 1.21 (1.09, 1.35) | 1.17 (1.05, 1.31) | 1.21 (1.09, 1.35) |
| **Frailty (criteria count; beta estimate with 95% CI)** | | | |
| DM9 | 0.14 (0.07, 0.20) | 0.10 (0.03, 0.17) | 0.14 (0.07, 0.20) |
| DM17 | 0.19 (0.12, 0.25) | 0.16 (0.09, 0.23) | 0.19 (0.12, 0.25) |
| DM31 | 0.20 (0.13, 0.26) | 0.17 (0.10, 0.23) | 0.20 (0.13, 0.26) |
| Allostatic load | 0.17 (0.11, 0.23) | 0.17 (0.10, 0.24) | 0.17 (0.11, 0.23) |
| SAH | 0.43 (0.37, 0.49) | 0.38 (0.32, 0.45) | 0.42 (0.36, 0.49) |
| **CVD (yes/no, odds ratio with 95% CI)** | | | |
| DM9 | 1.05 (0.73, 1.51) | 0.97 (0.74, 1.40) | 0.98 (0.70, 1.46) |
| DM17 | 1.85 (1.06, 3.08) | 1.64 (1.07, 2.60) | 1.62 (1.06, 2.49) |
| DM31 | 2.34 (1.55, 3.63) | 2.15 (1.30, 3.21) | 2.54 (1.59, 4.25) |
| Allostatic load | 0.57 (0.42, 0.84) | 0.57 (0.37, 0.83) | 0.58 (0.40, 0.85) |
| SAH | 1.37 (0.98, 1.81) | 1.38 (0.99, 1.86) | 1.39 (1.02, 1.94) |
| **Diabetes (yes/no, odds ratio with 95% CI)** | | | |
| DM9 | 0.75 (0.45, 1.11) | 0.65 (0.44, 0.99) | 0.77 (0.52, 1.23) |
| DM17 | 1.30 (0.78, 2.21) | 1.08 (0.63, 1.76) | 1.36 (0.76, 2.28) |
| DM31 | 2.98 (1.68, 5.90) | 2.81 (1.52, 5.30) | 3.73 (2.02, 7.26) |
| Allostatic load | 2.28 (1.38, 3.50) | 2.20 (1.51, 3.15) | 2.03 (1.44, 2.90) |
| SAH | 1.41 (0.99, 2.03) | 1.39 (0.92, 2.08) | 1.62 (1.09, 2.45) |
| **Comorbidity count (beta estimate with 95% CI)** | | | |
| DM9 | 0.08 (0.04, 0.11) | 0.08 (0.04, 0.11) | 0.07 (0.03, 0.11) |
| DM17 | 0.12 (0.02, 0.20) | 0.11 (0.07, 0.15) | 0.16 (0.11, 0.21) |
| DM31 | 0.22 (0.17, 0.26) | 0.20 (0.15, 0.25) | 0.20 (0.14, 0.28) |
| Allostatic load | 0.10 (0.07, 0.14) | 0.06 (0.01, 0.10) | 0.08 (0.04, 0.13) |
| SAH | 0.10 (0.05, 0.16) | 0.09 (0.06, 0.12) | 0.10 (0.05, 0.14) |

^a^ Model 1controls for age and sex.

^b^ Model 2 controls for age, sex, and metrics of physical and cognitive functions.

^c^ Model 3 controls for age, sex, and socio-economic status.

**Supplementary Table 4. Relationships between health metrics and various health outcomes in the WHAS dataset.**

|  | Model 1^a^ | Model 2^b^ | Model 3^c^ |
| --- | --- | --- | --- |
| **Mortality (hazard ratio with 95% CI)** | | | |
| DM9 | 1.27 (1.14, 1.41) | 1.30 (1.11, 1.52) | 1.21 (1.06, 1.39) |
| DM17 | 1.32 (1.19, 1.46) | 1.31 (1.13, 1.53) | 1.36 (1.19, 1.55) |
| DM31 | 1.21 (1.13, 1.30) | 1.37 (1.16, 1.62) | 1.24 (1.13, 1.36) |
| Allostatic load | 1.32 (1.19, 1.45) | 1.26 (1.08, 1.46) | 1.38 (1.21, 1.56) |
| SAH | 1.49 (1.33, 1.65) | 1.43 (1.21, 1.69) | 1.45 (1.25, 1.68) |
| **Frailty (criteria count; beta estimate with 95% CI)** | | | |
| DM9 | 0.11 (0.08, 0.15) | 0.05 (0.00, 0.10) | 0.09 (0.04, 0.14) |
| DM17 | 0.14 (0.10, 0.18) | 0.04 (0.00, 0.08) | 0.11 (0.07, 0.15) |
| DM31 | 0.16 (0.12, 0.20) | 0.04 (0.00, 0.10) | 0.15 (0.09, 0.19) |
| Allostatic load | 0.15 (0.11, 0.18) | 0.14 (0.09, 0.19) | 0.11 (0.07, 0.14) |
| SAH | 0.37 (0.33, 0.41) | 0.20 (0.15, 0.25) | 0.30 (0.25, 0.36) |
| **CVD (yes/no, odds ratio with 95% CI)** | | | |
| DM9 | 1.08 (0.96, 1.21) | 1.00 (0.85, 1.18) | 0.98 (0.84, 1.13) |
| DM17 | 1.19 (1.06, 1.34) | 1.07 (0.90, 1.26) | 1.09 (0.94, 1.27) |
| DM31 | 1.13 (1.01, 1.27) | 1.01 (0.86, 1.20) | 1.05 (0.91, 1.22) |
| Allostatic load | 1.19 (1.06, 1.33) | 1.17 (1.00, 1.37) | 1.19 (1.02, 1.38) |
| SAH | 1.88 (1.06, 1.33) | 1.75 (1.48, 2.09) | 2.01 (1.69, 2.39) |
| **Diabetes (yes/no, odds ratio with 95% CI)** | | | |
| DM9 | 1.06 (0.91, 1.25) | 0.92 (0.74, 1.13) | 0.88 (0.70, 1.09) |
| DM17 | 1.22 (1.04, 1.43) | 0.98 (0.78, 1.22) | 1.01 (0.81, 1.24) |
| DM31 | 2.18 (1.83, 2.61) | 1.98 (1.58, 2.52) | 1.93 (1.55, 2.43) |
| Allostatic load | 1.93 (1.65, 2.27) | 1.79 (1.46, 2.21) | 1.80 (1.46, 2.23) |
| SAH | 2.11 (1.77, 2.53) | 1.82 (1.45, 2.32) | 2.11 (1.66, 2.72) |
| **Comorbidity count (beta estimate with 95% CI)** | | | |
| DM9 | 0.05 (0.00, 0.10) | 0.01 (-0.06, 0.08) | 0.00 (-0.06, 0.07) |
| DM17 | 0.10 (0.05, 0.15) | 0.03 (-0.04, 0.10) | 0.06 (-0.01, 0.12) |
| DM31 | 0.16 (0.11, 0.20) | 0.12 (0.05, 0.19) | 0.14 (0.08, 0.20) |
| Allostatic load | 0.15 (0.10, 0.20) | 0.12 (0.06, 0.19) | 0.15 (0.08, 0.21) |
| SAH | 0.39 (0.34, 0.45) | 0.33 (0.26, 0.40) | 0.42 (0.35, 0.50) |

^a^ Model 1controls for age.

^b^ Model 2 controls for age and metrics of physical and cognitive functions.

^c^ Model 3 controls for age and socio-economic status.

1. **Supplementary Figures**

**
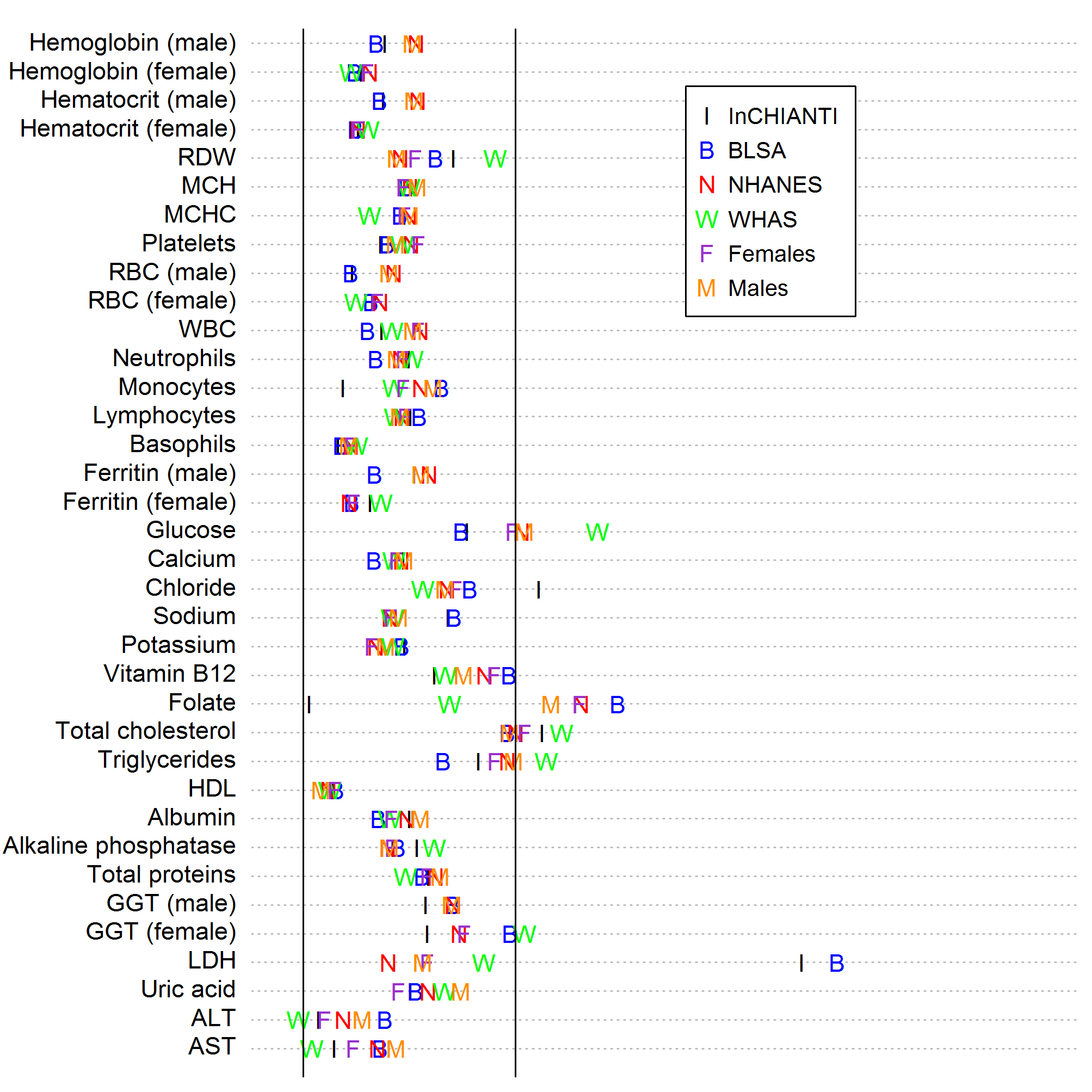
 Supplementary Figure 1.** Mean values for each biomarker at first visit were normalized according to the reported minimal and maximal normal values (see Supplementary Table 1), represented by the vertical lines. For biomarkers with only one specified normal value, the other vertical line represents minimal or maximal value of all datasets. Results are presented in black for InCHIANTI (“I”), in blue for BLSA (“B”), in red for NHANES (“N”), in green for WHAS (“W”), in purple for females (“F”), and in orange for males (“M”).

**
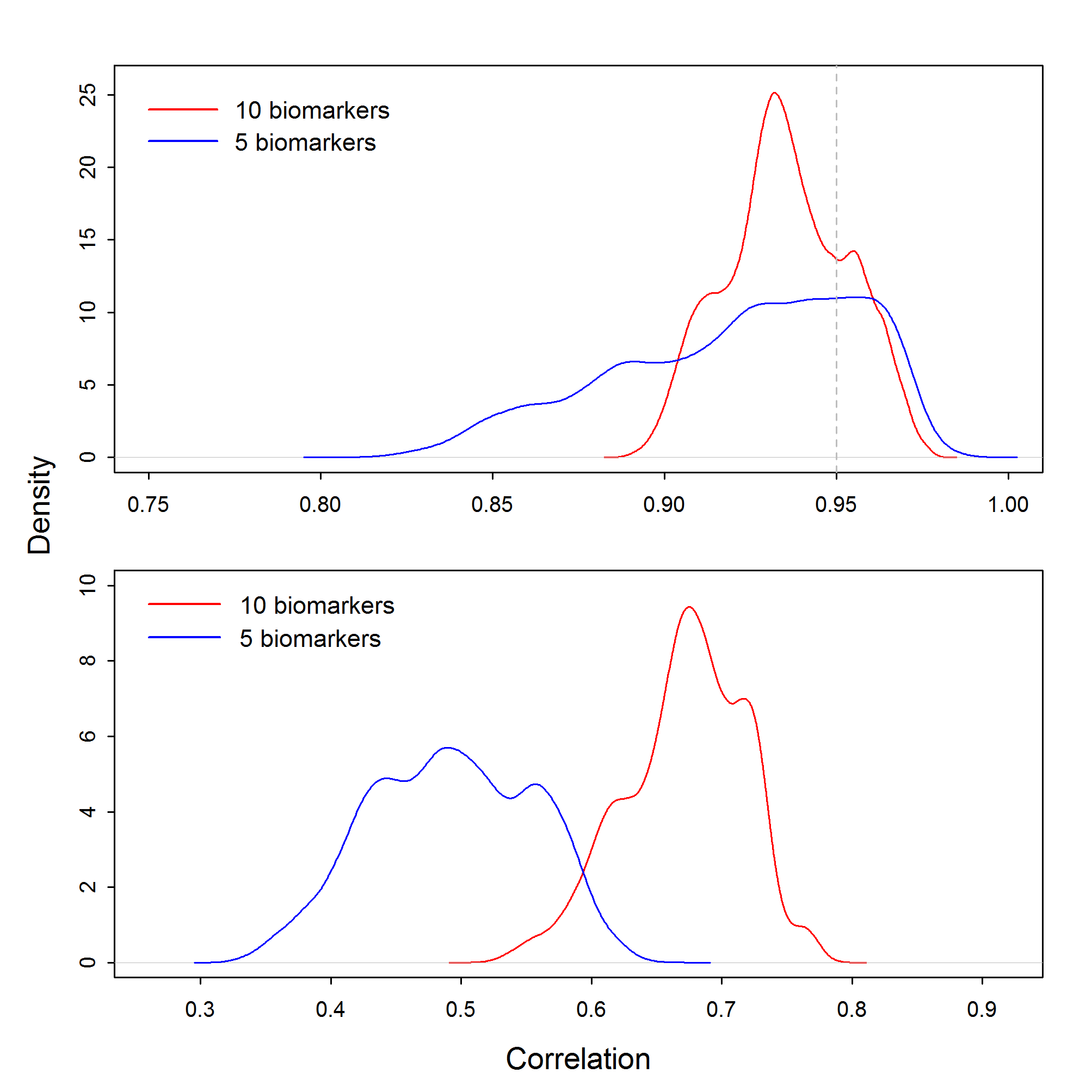
**

**Supplementary Figure 2.** *Upper panel*: density plot of correlations between DM calculated with a composite RP (composed of 1138 individuals from each of the three study datasets), with DM calculated with each dataset as its own RP, for each suite of five (blue) and ten (red) biomarkers among the 22-set. Frequencies of each biomarker among the combinations with a correlation coefficient >= 0.95 (indicated by the vertical dashed line) were then used for the selection of final sets. *Lower panel*: density plot of correlations between DM calculated using the full set (31 biomarkers) and each suite of five (blue) and ten (red) biomarkers among the 22-set.


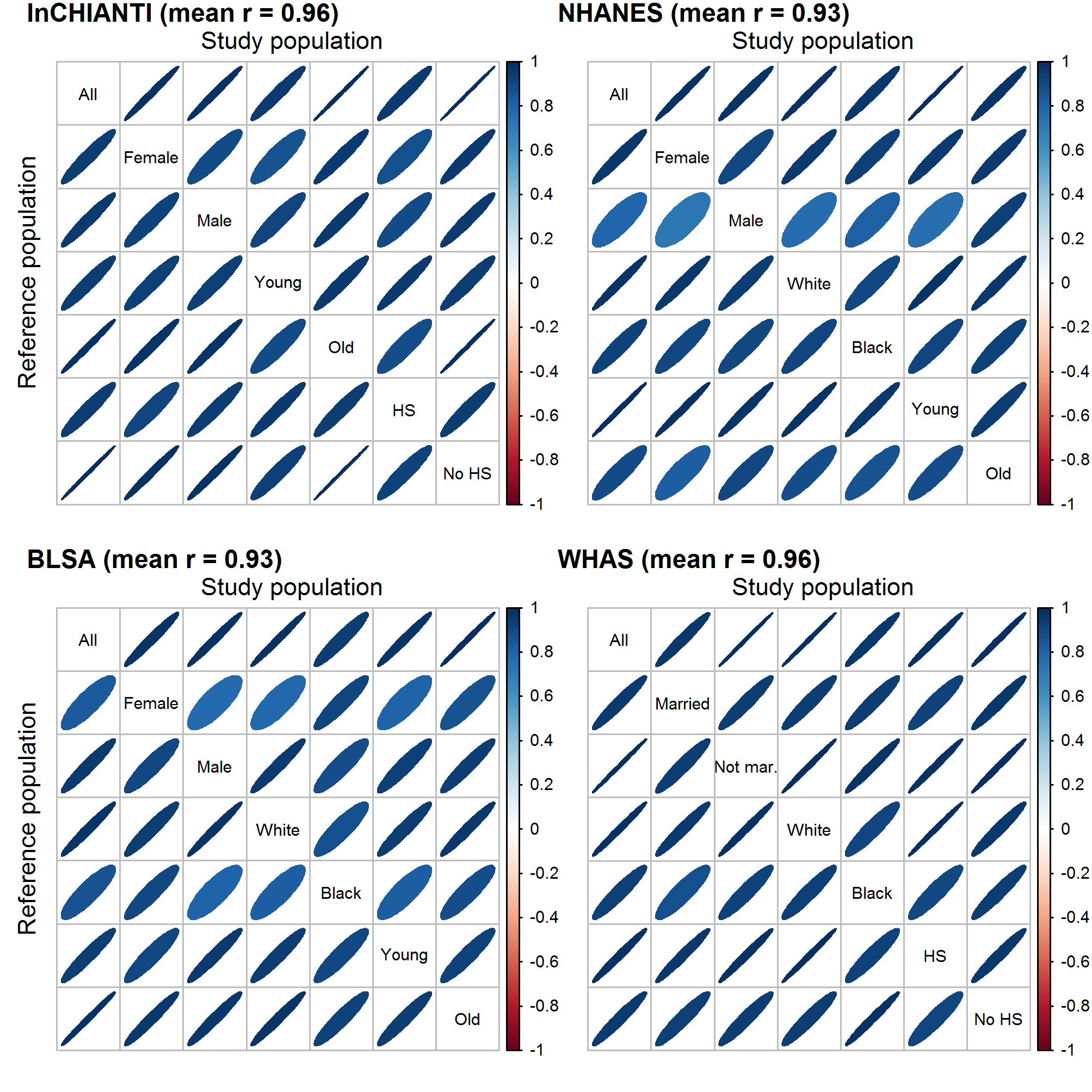


**Supplementary Figure 3. Stability of the 31 biomarker-set *D_M_* across different definitions of the reference population (RP).** For various demographic subsets of the population, we computed Pearson correlations between DM calculated using this particular subset as its RP (column) or another subset (lines). Correlations were calculated in all datasets: InCHIANTI, NHANES, BLSA, and WHAS. Mean correlation coefficients (r) are indicated above each dataset and ellipses indicate correlations visually, i.e. darker and narrower when stronger. Abbreviations: HS, high school diploma; No HS, no high school diploma; NM, not married.


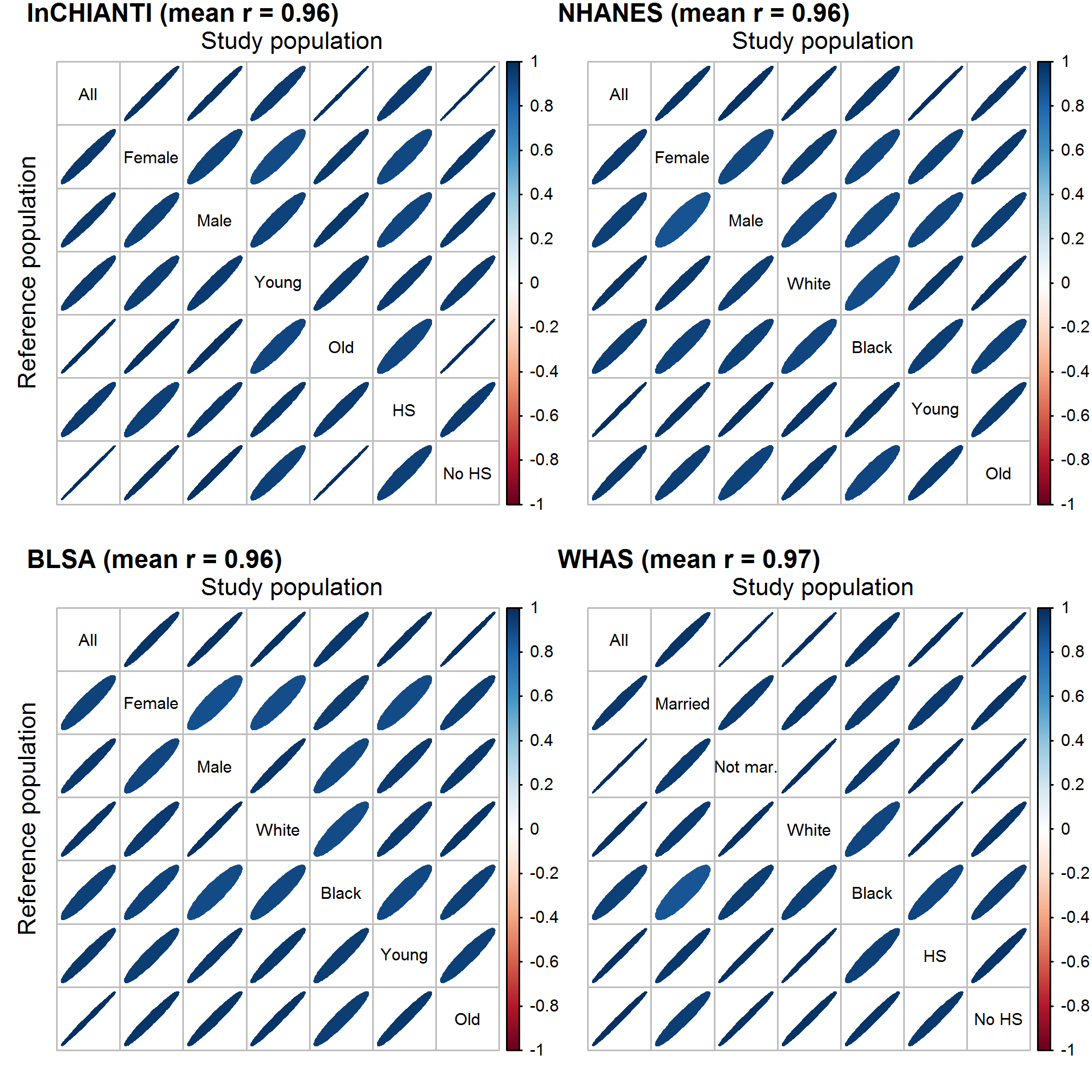


**Supplementary Figure 4. Stability of the 17 biomarker-set *D_M_* across different definitions of the reference population (RP).** For various demographic subsets of the population, we computed Pearson correlations between DM calculated using this particular subset as its RP (column) or another subset (lines). Correlations were calculated in all datasets: InCHIANTI, NHANES, BLSA, and WHAS. Mean correlation coefficients (r) are indicated for each dataset and ellipses indicate correlations visually, i.e. darker and narrower when stronger. Abbreviations: HS, high school diploma; No HS, no high school diploma; NM, not married.


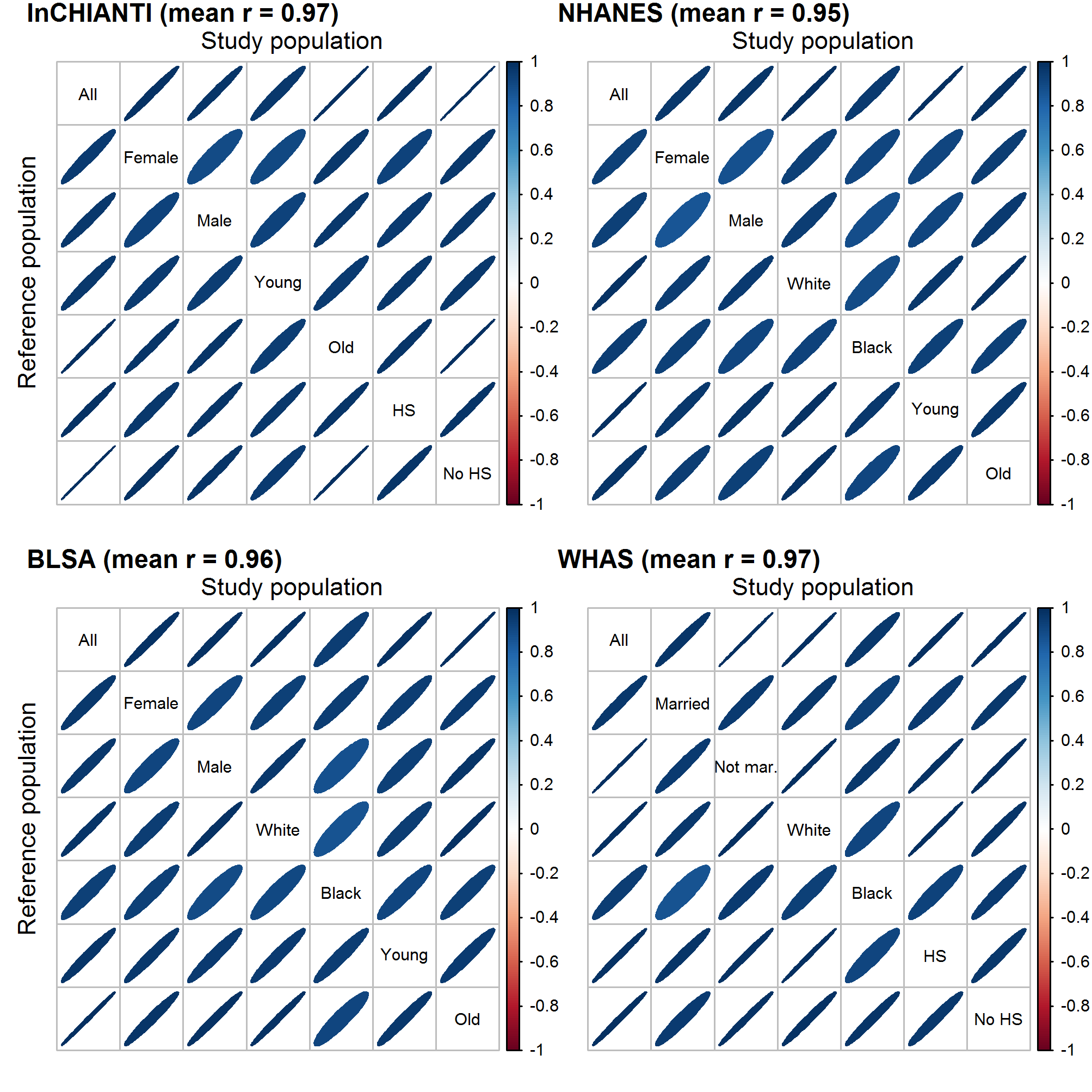


**Supplementary Figure 5. Stability of the 9 biomarker-set *D_M_* across different definitions of the reference population (RP).** For various demographic subsets of the population, we computed Pearson correlations between DM calculated using this particular subset as its RP (columns) or another subset (lines). Correlations were calculated in all datasets: InCHIANTI, NHANES, BLSA, and WHAS. Mean correlation coefficients (r) are indicated for each dataset and ellipses indicate correlations visually, i.e. darker and narrower when stronger. Abbreviations: HS, high school diploma; No HS, no high school diploma; NM, not married.


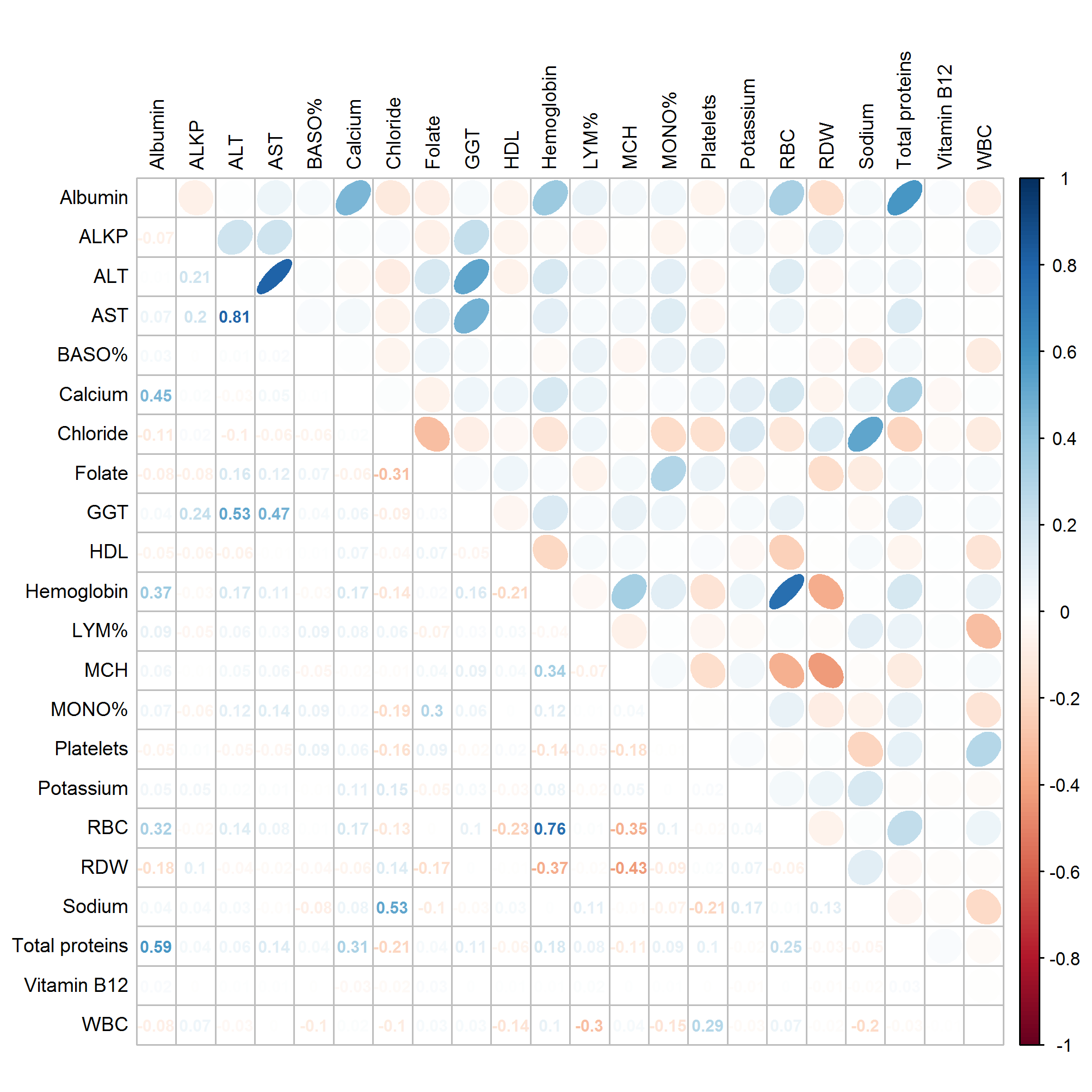


**Supplementary Figure 6.** Correlations between all biomarkers among the 22-set. Pearson correlations were calculated with an equal number (n = 1138) of individuals from the three study cohorts (BLSA, InCHIANTI, and NHANES). Ellipses above the diagonal indicate correlations visually: blue when positive, red when negative, and darker and narrower when stronger. Correlation coefficients are shown below the diagonal.
